# A temporally functional composite hydrogel scaffold for cranial defect repair via sequential modulation of angiogenesis and osteogenesis

**DOI:** 10.7150/thno.119835

**Published:** 2026-01-01

**Authors:** Zongqiang Lv, Bo Sun, Rong Li, Bowen Zhao, Hongxiang Wang, Ning Luo, Xin Ding, Xuan Tang, Chunlin Wang, Long Bai, Jiacan Su, Juxiang Chen

**Affiliations:** 1 Department of Neurosurgery, The First Affiliated Hospital of Naval Medical University, Shanghai, 200433, China.; 2 Department of Neurosurgery, No.901 Hospital of Joint Logistics Support Force of PLA, Hefei, 230031, Anhui, China.; 3 Organoid Research Center, Institute of Translational Medicine, Shanghai University, Shanghai, 200444, China.; 4 National Center for Translational Medicine (Shanghai) SHU Branch, Shanghai University, Shanghai, 200444, China.; 5 Department of Orthopedics, Xinhua Hospital Affiliated to Shanghai Jiao Tong University School of Medicine, Shanghai, 200092, China.; 6 MedEng-X Institutes, Shanghai University, Shanghai, 200444, China.

**Keywords:** silk fibroin hydrogel, salvianolic acid B, mineralized hydrogel microsphere, cranial defect, temporal regulation

## Abstract

The repair of large cranial defects remains a major clinical challenge, as conventional materials primarily act as inert fillers and fail to meet the complex biological requirements of cranial bone regeneration. In particular, they lack the ability to temporally coordinate angiogenesis and osteogenesis. This study aimed to develop a temporally functional composite scaffold to dynamically modulate the regenerative microenvironment and promote sequential vascularized bone regeneration.

**Methods:** A silk fibroin-based hydrogel system was designed, incorporating salvianolic acid B (SalB)-loaded sustained-release hydrogel and mineralized silk fibroin hydrogel microspheres (MSFM). Material characterization was performed to evaluate the structural and mechanical properties of the scaffold, as well as the drug release behavior. *In vitro* assays were conducted to assess endothelial cell migration, tube formation, and the expression of angiogenesis-related genes, along with the osteogenic differentiation potential of bone marrow-derived mesenchymal stem cells (BMSCs). *In vivo* reparative efficacy was further validated using a rat cranial defect model through morphological and histological analyses.

**Results:** Characterization confirmed that OSFM microgels were uniformly spherical with a porous internal structure and exhibited sustained release of OGP. *In vitro*, OSFM showed excellent cytocompatibility with BMSCs, significantly enhancing cell proliferation, ALP activity, and mineralized nodule formation compared with SFM (p < 0.05). Tube formation and scratch assays demonstrated that OSFM-conditioned medium promoted HUVEC migration and angiogenesis. *In vivo*, implantation of OSFM+PCL scaffolds into rat calvarial defects resulted in markedly superior bone regeneration compared with control, PCL, and SFM+PCL groups. The bone volume fraction in the OSFM+PCL group reached 52.31 ± 4.27% at the 8th weeks, significantly higher than 23.65 ± 3.81%, 30.42 ± 3.96%, and 37.86 ± 4.12% in the other groups (p < 0.05). Histological staining confirmed more mature bone formation, abundant collagen deposition, and tight integration between new bone and scaffold. Immunohistochemistry revealed upregulated expression of RUNX2, OCN, and CD31, indicating enhanced osteogenesis and angiogenesis.

**Conclusions:** This temporally functional composite scaffold achieved a sequential “angiogenesis first, osteogenesis later” strategy by leveraging the differential degradation kinetics of its components. The findings demonstrate a biomimetic and temporally regulated approach with strong bioactivity and translational potential for cranial bone regeneration.

## Introduction

Cranial bone defects arising from traumatic brain injury, tumor resection, or cerebral hemorrhage pose significant clinical challenges 1. Such defects compromise the mechanical integrity of the cranial cavity and may cause neurological impairments and craniofacial deformities, leading to a marked decline in quality of life 2. Conventional repair materials, such as titanium mesh and polyetheretherketone (PEEK), primarily restore structural integrity but fail to recapitulate the dynamic biological processes essential for natural bone regeneration 3, 4.

Bone regeneration is a multistage process that requires precise coordination to achieve tissue repair and functional restoration 5, 6. During the early phase, rapid neovascularization establishes networks for oxygen and nutrient supply and recruits osteoprogenitor cells, thereby supporting subsequent bone formation. In later stages, enrichment of calcium and phosphate ions within the local microenvironment synergizes with osteoblast differentiation to facilitate bone matrix deposition, ultimately restoring skeletal structure and function 7, 8. Increasing evidence indicates that activation of endothelial progenitor cells and coupling of angiogenesis with osteogenesis are critical for vascularization during bone repair 9, 10. Despite this, existing cranial repair materials predominantly rely on static designs and lack responsiveness to dynamic biological events such as neovascularization and sustained mineralization, resulting in suboptimal outcomes. Therefore, scaffolds that integrate angiogenic and mineralization functions present a more effective approach to enhance cranial bone regeneration 11.

Hydrogels have emerged as promising candidates in bone tissue engineering due to their cytocompatibility, tunable physicochemical properties, and adaptability to dynamic biological environments 12, 13. Silk fibroin-based hydrogels (SFH) possess distinct advantages owing to their unique hierarchical β-sheet crystalline structures 14, 15. This architecture imparts high mechanical strength and pronounced strain-stiffening behavior, enabling resistance to physiological loads at bone interfaces. SFH also demonstrates outstanding biocompatibility, supporting cellular adhesion, proliferation, and differentiation. Furthermore, the abundance of carboxyl and hydroxyl groups provides reactive sites for bioactive molecule binding and facilitates dynamic mineralization interfaces, promoting calcium and phosphate deposition for bone matrix formation. Compared with conventional hydrogels such as gelatin and alginate, SFH demonstrates superior mechanical performance and greater osteoinductive capacity 16. This dual functionality—mechanical robustness combined with intrinsic bioactivity-positions SFH as an ideal platform for cranial bone repair and regeneration.

Salvianolic acid B (SalB), a bioactive compound from *Salvia miltiorrhiza*, exhibits diverse pharmacological properties, including proangiogenic effects, but its clinical application is restricted by poor stability and limited bioavailability 17-19. Incorporation of SalB into hydrogels offers a strategy for sustained release, enhancing angiogenesis while providing a stable osteogenic microenvironment. Such controlled delivery facilitates blood vessel formation and concurrently supports bone tissue development 20-22.

Mineralized hydrogels, which recapitulate natural mineralization processes, improve osteoblast activity and mechanical stability 23. Incorporation of mineralized silk fibroin microspheres within SalB-loaded hydrogels creates a synergistic effect, providing nucleation sites and releasing calcium/phosphate ions to promote both angiogenesis and osteogenesis. This integration enhances scaffold performance by coupling vascularization with mineralization, thereby advancing regenerative efficacy.

Based on these considerations, a composite hydrogel scaffold with temporally regulated functionality is proposed. The system integrates low-concentration SFH with SalB to promote early angiogenesis, while mineralized SFH microspheres provide calcium and phosphate ions to sustain osteogenesis. Temporal regulation is achieved through controlled degradation of scaffold components, ensuring sequential coordination of angiogenesis and osteogenesis throughout the repair process.

## Materials and Methods

### Materials

Salvianolic acid B (SalB) was purchased from Sigma-Aldrich(USA). Silk fibroin methacrylate (SilMA) and lithium phenyl(2,4,6-trimethylbenzoyl) phosphinate (LAP) were purchased from Engineering for Life (Jiangsu, China). Phosphate-buffered saline (PBS) and 4% paraformaldehyde (PFA) were purchased from Servicebio (Hubei, China). Liquid paraffin and Span 80 were obtained from Aladdin (Shanghai, China). Sodium chloride (NaCl), potassium chloride (KCl), magnesium chloride hexahydrate (MgCl₂·6H₂O), calcium chloride dihydrate (CaCl₂·2H₂O), sodium dihydrogen phosphate (NaH₂PO₄), and sodium bicarbonate (NaHCO₃) were purchased from General-Reagent (Shanghai, China).

### Cell culture

Bone marrow mesenchymal stem cells (BMSCs) were cultured in α-Minimum Essential Medium (α-MEM; Corning), and human umbilical vein endothelial cells (HUVECs) were maintained in Dulbecco's Modified Eagle Medium (DMEM; Corning). Both media were supplemented with 10% fetal bovine serum (FBS; Sigma) and 100 U/mL penicillin-streptomycin (Gibco). All cell lines were procured from Cyagen Biosciences.

### Preparation of SilMA hydrogel microspheres

SilMA hydrogel microspheres were prepared using a microfluidic approach 24. The photoinitiator solution was prepared by dissolving 0.125 g LAP in 50 mL PBS and heating at 40-50 °C for 15 min under continuous stirring. SilMA was then dissolved in LAP solution to a final concentration of 15% (w/v) and stirred gently at room temperature for 30-60 min, followed by sterilization using 0.22 μm syringe-driven filter. The continuous phase consisted of liquid paraffin containing 10% (w/v) Span 80. The SilMA solution (dispersed phase) and paraffin mixture (continuous phase) were loaded into separate syringes connected to a microfluidic droplet generator. Monodisperse droplets were generated at flow rates of 10 μL/min (dispersed phase) and 100 μL/min (continuous phase), followed by ultraviolet (UV) crosslinking (405 nm, 10 mW/cm²). The resulting microspheres were collected, washed sequentially with PBS and 75% ethanol to remove residual oil and surfactant, and equilibrated in PBS for 4 h with four solvent exchanges.

### Mineralization of SilMA hydrogel microspheres

Mineralization was performed using a concentrated 10× simulated body fluid (SBF) solution prepared according to the Tas and Bhaduri method 25, 26. The 10× SBF stock solution was prepared by sequentially dissolving NaCl (116.886 g), KCl (0.7456 g), CaCl₂·2H₂O (7.3508 g), MgCl₂·6H₂O (2.033 g), and NaH₂PO₄ (2.3996 g) in 2000 mL deionized water, followed by storage at 4 ℃. Prior to mineralization, 40 mL of the stock solution was mixed with 33.6 mg NaHCO₃ to obtain a final HCO₃⁻ concentration of 10 mM (Table S1). Hydrogel microspheres were rinsed three times with deionized water, immersed in the mineralizing solution, and incubated at 37 ℃ under orbital shaking (100 rpm) for 60-240 min. Following mineralization, the microspheres were extensively washed with deionized water to eliminate weakly bound mineral residues.

### Preparation of SalB-SilMA composite hydrogel

The SalB-SilMA composite hydrogel (SalB@SFH) was synthesized via a photo-crosslinking strategy 27, 28. Briefly, 0.16 g of SilMA was dissolved in 100 μL LAP solution under gentle agitation at room temperature for 1 h to obtain a 16% (w/v) SilMA precursor. SalB powder was separately dissolved in 100 μL LAP solution at a predefined concentration and stored at 4 °C in amber centrifuge tubes to prevent photodegradation. The SalB solution was then gradually mixed with the SilMA precursor (final SilMA concentration: 8% w/v) under continuous cooling (4 °C) to ensure homogeneous distribution. The resulting composite precursor solution was exposed to UV light (405 nm, 60 s) to induce gelation and form stable SalB@SFH.

The effect of different SalB concentrations on HUVECs viability was evaluated by the Cell Counting Kit-8 (CCK-8). Hydrogel precursor solutions containing SalB at concentrations of 0 μM, 2 μM, 20 μM, 200 μM, and 2 mM were dispensed into 96-well plates. Following UV-induced crosslinking (405 nm, 60 s) and sterilization, SalB@SFH was rinsed three times with PBS. HUVECs were seeded onto the SalB@SFH surfaces at a density of 5 × 10³ cells/well and cultured for 72 h in cell incubator (37 °C, 5% CO₂). Subsequently, 110 μL of CCK-8 reagent diluted in fresh medium (10% v/v) was added to each well. After 1 h of incubation, the optical density was measured at 450 nm.

### Assembly of temporally functionalized hydrogel composite scaffolds

Composite hydrogel scaffolds were fabricated by integrating MSFM with SalB@SFH. Pre-mineralized microspheres were packed into cylindrical molds, and excess solution was removed by gentle aspiration. The SalB-SilMA precursor solution (8% w/v) was introduced to infiltrate the microspheres completely, followed by UV crosslinking (405 nm, 10 mW/cm², 30 s) to yield a cohesive scaffold.

### Characterization of temporally functionalized hydrogel composite scaffolds

#### Microsphere morphology and particle size analysis

Microsphere morphology and particle size were assessed by dispersing the samples in PBS and observing them using optical and brightfield microscopy. Images were captured, and particle size distributions of non-mineralized and mineralized microspheres were analyzed using ImageJ software. Microstructure analysis of microspheres and hydrogels was performed by freezing the samples at -20 °C for 12 h, followed by lyophilization for 48 h. The dehydrated specimens were mounted on sample stubs, coated with a thin gold layer via sputter deposition, and examined by scanning electron microscopy (SEM). Elemental distribution scanning was performed on MSFM to determine elemental composition.

#### Mechanical properties of microspheres

The mechanical properties of non-mineralized and mineralized microspheres were assessed using a Microtester microforce testing instrument. Force-displacement curves were generated, and data were analyzed using GraphPad Prism software.

#### Rheological properties of hydrogels

The rheological properties of SFH and SalB@SFH were measured using a rotational rheometer. Samples with smooth, bubble-free surfaces were prepared. Measurements were conducted at 25 ℃ under a constant strain of 5%, with angular frequencies ranging from 100 rad/s to 0.1 rad/s. The storage modulus (Gʹ) and loss modulus (Gʺ) were recorded to characterize viscoelastic behavior.

#### Swelling and degradation behavior

For swelling experiments, dried hydrogel microspheres and bulk hydrogels with smooth, bubble-free surfaces were weighed to obtain the initial dry weight (*W_0_*). Samples were immersed in PBS at room temperature in a shaking incubator. At the designated time points, samples were collected, surface moisture was removed, and the weight was recorded (Wₜ). The swelling ratio was calculated as:



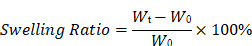



For degradation experiments, samples were prepared as described above, and the initial dry weight (*W_0_*) was recorded. Samples were then immersed in either PBS or proteinase XIV solution (1 mg/mL) and incubated at room temperature under shaking (100 rpm). The degradation medium was refreshed every 48 h to maintain enzyme activity. At designated time points, samples were collected, blotted to remove surface moisture, and weighed (*W_t_*). The percentage of remaining mass was calculated as:



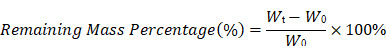



Degradation behavior was expressed as mass-retention curves over time.

#### Release behavior of SalB from SalB@SFH

A standard calibration curve for SalB quantification was generated by preparing SalB solutions at defined concentrations (5 μM, 10 μM, 20 μM, 40 μM, 80 μM, and 400 μM), and their absorbance was measured at 286 nm using a UV spectrophotometer. Absorbance values were plotted against concentration to establish a linear regression curve. SalB-loaded silk fibroin composite hydrogels were prepared with uniform distribution, and both the weight of the hydrogels and the initial SalB loading were recorded. Samples were fully immersed in PBS in centrifuge tubes and incubated at room temperature under shaking (100 rpm). At specified time points, 1 mL of supernatant was collected and analyzed for absorbance at 286 nm. The concentration of SalB was determined from the absorbance values using a standard calibration curve. After each sampling, 1 mL of fresh PBS was added to maintain a constant total volume. The cumulative release of SalB was then calculated and presented as a release profile over time.

### Biocompatibility evaluation of composite scaffolds

The biocompatibility of composite scaffolds was assessed by co-culturing BMSCs and HUVECs in direct contact with scaffold materials. Experimental groups included: control (cells only), silk fibroin hydrogel (SFH), SalB-loaded hydrogel (SalB@SFH), SalB-loaded hydrogel with microspheres (SalB@SFH+SFM), and SalB-loaded hydrogel with mineralized microspheres (SalB@SFH+MSFM). For hydrogel-containing groups, defined amounts of hydrogels or microspheres were added to wells and crosslinked under UV light (405 nm, 60 s). All hydrogel samples were sterilized by UV irradiation before use.

BMSCs and HUVECs were seeded at a density of 5 × 10³ cells per well and cultured for 1, 3, and 5 days. At each time point, the culture medium was removed, the wells were washed twice with PBS, and subsequently supplemented with 90 μL of fresh medium containing 10 μL of CCK-8 reagent. Samples were incubated for 1 h at RT, and absorbance was measured at 450 nm to determine cell viability.

Live/dead staining was performed to further evaluate scaffold cytocompatibility. BMSCs and HUVECs were seeded at 1 × 10^5^ cells/well onto confocal dishes containing the respective scaffold components. After 48 h of incubation, the medium was aspirated, and samples were rinsed with PBS. Calcein-AM/propidium iodide (PI) staining solution was then added and incubated at room temperature for 30 min. Confocal laser scanning microscopy was employed to visualize cellular morphology, viability, and spatial distribution on the hydrogel scaffolds.

### Evaluation of angiogenic activity of composite hydrogel scaffolds *in vitro*

The angiogenic potential of composite hydrogel scaffolds was assessed through scratch wound assays and tube formation assays. A Transwell system was employed to maintain indirect contact between scaffolds and HUVECs cultured at the bottom of the wells, preventing direct interference with the observation field. Experimental groups consisted of control, SFH, SalB@SFH, SalB@SFH+SFM, and SalB@SFH+MSFM. Each group was tested in triplicate (n = 3).

#### Cell migration assay

HUVECs were cultured to 80-90% confluence, after which a linear scratch was introduced across the cell monolayer using a sterile 200 μL pipette tip. The wells were gently rinsed to remove detached cells, and fresh culture medium supplemented with 2% serum was subsequently added. Transwell inserts containing the corresponding scaffold formulations were placed in each well. After 24 h of incubation, wound closure was observed and imaged using an inverted microscope to evaluate cell migration.

#### Tube formation assay

Matrigel was thawed on ice and added to pre-cooled 24-well plates to form a uniform gel layer. HUVECs were seeded on Matrigel at a density of 1 × 10⁵ cells per well, and Transwell inserts containing the scaffold components were introduced. After Calcein staining, tube formation was evaluated at 4 h and 8 h. Vascular network structures were visualized under a fluorescence microscope, and tubes numbers were quantified to compare angiogenic activity among groups. Each condition was tested in triplicate (n = 3).

### Evaluation of osteogenic activity of composite hydrogel scaffolds *in vitro*

The osteogenic differentiation potential of temporally functionalized composite hydrogel scaffolds was assessed using alkaline phosphatase (ALP) staining and Alizarin Red S (ARS) staining. Experimental groups included: control, SFH, SalB@SFH, SalB@SFH+SFM, and SalB@SFH+MSFM. For the SFH and SalB@SFH groups, defined amounts of precursor solution were dispensed into wells and photo-crosslinked with UV light (405 nm, 30 s) to form hydrogels, followed by UV sterilization. For the SalB@SFH+SFM and SalB@SFH+MSFM groups, specified amounts of SFM or MSFM were introduced into wells, after which SalB@SFH precursor solution was added to completely immerse the microspheres. The resulting mixture was photo-crosslinked with UV light (405 nm) and subsequently sterilized before use.

#### ALP staining assay

Pre-prepared 24-well plates containing the designated hydrogel conditions were seeded with BMSCs at a density of 2 × 10⁴ cells/well. Cells were cultured in α-MEM until reaching 60-70% confluence, after which the medium was replaced with osteogenic induction medium and renewed every 48 h to promote osteogenic differentiation. After 7 days of induction, samples were fixed with 4% paraformaldehyde for 15 min at room temperature and washed three times with PBS (5 min each). ALP staining was performed using a BCIP/NBT colorimetric kit. The working solution was added to each well and incubated at room temperature for 30 min, followed by gentle rinsing with PBS to remove residual dye. Images were captured, and semi-quantitative analysis was performed to assess osteogenic differentiation.

#### ARS staining assay

Following the same cell culture and osteogenic induction protocol as described for ALP staining, cells were induced for 14 days. Samples were then fixed and washed twice with distilled water before the addition of 2% ARS solution. After 30 min of incubation at room temperature, unbound dye was removed. Images were captured, and semi-quantitative analysis was conducted to assess calcium deposition.

### Related gene expression analysis

#### Quantitative real-time polymerase chain reaction (RT-qPCR)

BMSCs and HUVECs were cultured in the previously prepared plates under the following experimental conditions: Control (cells only), SFH, SalB@SFH, SalB@SFH+SFM, and SalB@SFH+MSFM. In the Control group, no materials were added, while in the other groups precursor solutions were crosslinked by UV light to form hydrogels. HUVECs were seeded at 2 × 10⁵ cells/well, and total RNA was extracted after 3 days of culture. Quantitative RT-PCR was conducted to assess angiogenesis-related genes, including *HIF-α, VEGF, ANG-1, eNOS*, and *FGF*, using *GAPDH* as the reference gene. Similarly, BMSCs were seeded in osteogenic induction medium, and RNA was extracted after 7 days. Osteogenesis-related gene expression was analyzed, including *ALP, COL1, BMP-2, RUNX2,* and *OCN*. The primer sequences used are listed in Table S2.

#### Transcriptome sequencing

For transcriptomic analysis, BMSCs and HUVECs were cultured under three conditions: Control, SalB@SFH, and SalB@SFH+MSFM. RNA was extracted from each group and subjected to high-throughput sequencing. Differentially expressed RNAs related to angiogenesis and osteogenesis were identified. Functional annotation was carried out using Gene Ontology (GO) enrichment, Kyoto Encyclopedia of Genes and Genomes (KEGG) pathway, and Gene Set Enrichment Analysis (GSEA). Genes with |log_2_(fold change)| ≥ 1 and false discovery rate (FDR) < 0.05 were considered significant and included in subsequent analyses.

### *In vivo* experiments

#### Rat cranial bone defect model

All animal procedures complied with the guidelines and were approved by the Ethics Committee of Shanghai University (ECSHU 2024-127). Sprague-Dawley rats (female, 5-6 weeks old) were used to establish a bilateral calvarial defect model. Rats were randomly divided into five groups: Control, SFH, SalB@SFH, SalB@SFH+SFM, and SalB@SFH+MSFM (n = 6 per group). Each group received implantation of the corresponding hydrogel composite material.

General anesthesia was induced via intraperitoneal injection of sodium pentobarbital (30 mg/kg). Adequate anesthesia was confirmed by the absence of corneal reflex and lack of response to nociceptive stimuli. Rats were placed in a prone position and secured in a stereotaxic frame. The surgical site was shaved, disinfected, and draped under sterile conditions.

A 3 cm midline incision was made to expose the parietal bone. Symmetrical round calvarial defects (5 mm in diameter) were created on either side of the sagittal suture using a low-speed cranial drill (250 rpm) under continuous irrigation with sterile saline to prevent thermal injury. Bone debris was removed, and the dura mater was carefully inspected to confirm integrity and hemostasis.

Hydrogel composite materials prepared according to group assignment were implanted into the defects, and incisions were closed with surgical sutures. At 4 and 8 weeks post-surgery, rats were euthanized, and calvarial samples were harvested. The skulls containing defect regions were excised with surgical scissors, leaving the periosteum intact and removing excess soft tissue. Specimens were fixed in 4% paraformaldehyde for 48 h before analysis.

#### Micro-CT and histological analysis

Excised cranial samples were scanned using a micro-computed tomography (Micro-CT) system (Skyscan 1176, Bruker), and three-dimensional reconstructions were generated. Quantitative morphometric parameters, including new bone volume, trabecular number, trabecular thickness, and bone mineral density, were analyzed to evaluate bone regeneration.

Histological analyses were performed on decalcified sections using hematoxylin and eosin (H&E) staining and Masson's trichrome staining to evaluate tissue morphology and collagen deposition. Immunohistochemistry and immunofluorescence were conducted to localize and assess the expression of angiogenic and osteogenic markers in regenerated tissues.

#### *In vivo* biocompatibility

To evaluate systemic biocompatibility, major organs (heart, liver, spleen, lung, and kidney) were collected from scaffold-implanted rats. H&E staining was performed to examine histological architecture and assess potential toxicological effects.

### Statistical analysis

Statistical analyses were performed using GraphPad Prism 10.1 (GraphPad Software, Inc.). Data are presented as mean ± standard deviation (SD) from at least three independent experiments. Group differences were analyzed using one-way or two-way analysis of variance (ANOVA), followed by appropriate post hoc multiple-comparison tests. Statistical significance was defined as *p < 0.05, **p < 0.01, ***p < 0.001, and ****p < 0.0001.

## Results and Discussion

### Preparation and characterization of SFM and MSFM

**Figure 2:**
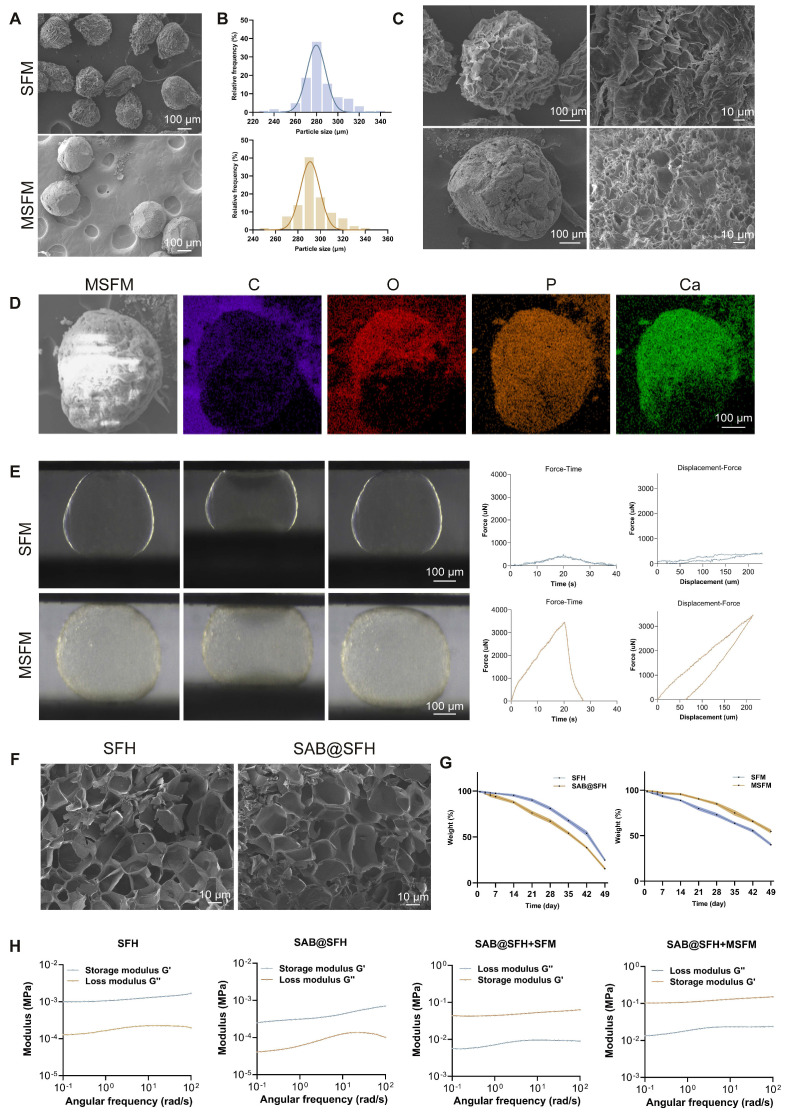
Synthesis and characterization of the temporally functional composite hydrogel scaffold. (A-C) SEM images and particle size analysis of SFM and MSFM. Scale bars = 100 μm and 10 μm. (D) EDS mapping of MSFM. Scale bar = 50 μm. (E) Compression-displacement curves of SFM and MSFM. Scale bar = 100 μm. (F) SEM images of SFH and SalB@SFH. Scale bar = 10 μm. (G) Degradation profiles of SFH and SalB@SFH. (H) Rheological curves of SFH, SalB@SFH, SalB@SFH+SFM, and SalB@SFH+MSFM.

SFH microspheres were synthesized using microfluidic technology, and their morphology was examined by optical microscopy. The microspheres displayed a spherical shape with uniform size distribution and an average diameter of 279.6 ± 8.8 μm (Figure 2B; Figure S1A), confirming the capacity of microfluidics to precisely control microsphere geometry. Mineralized SFH microspheres (MSFM) were subsequently prepared by immersing the microspheres in 10× simulated body fluid (SBF). Optical microscopy showed that mineralized microspheres exhibited reduced transparency while maintaining regular spherical morphology, with a slightly larger diameter of 290.9 ± 7.6 μm (Figure 2B). The increase in size indicated that mineralization modestly expanded the microsphere structure. SEM revealed that SFM had a loose, porous surface, whereas the MSFM displayed uniform mineral-like deposits covering their surface (Figures 2A, C). Time-dependent mineralization studies revealed a progressive increase in surface deposits, most prominent within the first 2 h, after which deposition reached a plateau at 3 h (Figure S2). Based on above results, a 3-hour period for mineralization was selected for the following experiments. EDS results indicated that, in addition to carbon (C) and oxygen (O), calcium (Ca) and phosphorus (P) were evenly distributed across the surface of mineralized microspheres (Figure 2D). The Ca/P mass ratio of 2.06 (Figure S3), consistent with the ratio characteristic of natural bone, suggesting the deposits were primarily calcium phosphate salts. Mineralization refers to the formation of calcium phosphate or hydroxyapatite deposits form on a biomaterial surface or within its structure, mimicking the mineralization observed in natural bone tissue. This biomimetic mineral layer provides a bone matrix-like environment that supports osteoblast adhesion, proliferation, differentiation, and matrix formation, thereby promoting osteogenesis. Mineralization also enhances the mechanical properties of the hydrogel, improving hardness and rigidity to levels more comparable to those of natural bone 29. SBF, an aqueous solution with ionic composition similar to human plasma, has been widely used to induce mineral deposition on biomaterials 30. While mineralization with conventional SBF is relatively slow, the use of 10× SBF, with tenfold higher ion concentration, accelerates calcium phosphate nucleation and growth, enabling rapid and efficient microsphere mineralization 25. This accelerated process is advantageous for fabricating osteoinductive biomaterials such as MSFM.

Mechanical testing further confirmed the effect of mineralization on microsphere performance. Force-displacement curves demonstrated that non-mineralized microspheres deformed easily under compressive stress, consistent with low rigidity and elastic modulus (Figure 2E). In contrast, mineralized microspheres showed a steeper curve, with minimal deformation under stress, indicating enhanced stiffness and elastic modulus. The force-displacement curve of non-mineralized microspheres was symmetrical, suggesting a uniform mechanical response and greater ease of deformation, whereas the asymmetry observed in mineralized microspheres reflected enhanced rigidity and brittleness. This mechanical reinforcement is essential for evaluating scaffold stability and load-bearing capacity in practical applications, particularly under external pressure.

### Preparation and characterization of SalB@SFH

SalB@SFH hydrogels were prepared by incorporating SalB into the precursor solution, followed by UV-induced crosslinking. SEM analysis revealed that SFH displayed a uniform, regular porous structure with relatively small and evenly distributed pores, whereas SalB@SFH exhibited slightly larger and more irregular pores (Figure 2F). These morphological changes were attributed to the effect of SalB on hydrogel crosslinking density, which altered the microstructure.

The optimal SalB concentration in the composite hydrogel was determined using CCK-8 assays to evaluate HUVEC viability at varying SalB concentrations. Cell viability exhibited a concentration-dependent response, with proliferation increasing as SalB concentration increased. The OD value peaked at 200 μM, but decreased at higher concentrations (2 mM) (Figure S6). Previous studies have reported effective SalB concentrations in the range of 10-100 μM. Since SalB release from the hydrogel is gradual rather than immediate, the early effective concentrations are expected to be much lower than 200 μM, which is consistent with prior findings. Therefore, 200 μM was chosen for the working concentration for following experiments.

SalB release behavior was further evaluated to characterize its temporal profile and confirm its potential for early angiogenic stimulation. A standard calibration curve established by UV spectrophotometry showed a strong linear correlation between absorbance and concentration (R² = 0.998), providing a reliable basis for quantitative analysis (Figure S4A). Release studies revealed that SalB exhibited a sustained release profile (Figure S4B). During the initial 1-5 days, a rapid release phase was observed, likely due to hydrogel swelling, which facilitated SalB diffusion. This early burst ensured an adequate supply of bioactive molecules to promote angiogenesis. From days 6-10, the release rate gradually decreased, attributable to progressive hydrogel degradation and controlled diffusion, thereby maintaining stable and effective concentrations supportive of prolonged angiogenesis. In the later phase, release plateaued, indicating near-complete drug release. This sustained release property represents a critical feature of the composite hydrogel scaffold, enabling sequential functional regulation to meet the temporal requirements of angiogenesis and osteogenesis during cranial defect repair.

### Preparation and characterization of the composite hydrogel scaffold

The degradation behaviors of SalB@SFH, SFH, SFM, and MSFM were investigated to assess the sequential regulation of angiogenesis and osteogenesis. SalB@SFH exhibited the fastest degradation rate, facilitating rapid SalB release during the early phase of bone repair to promote angiogenesis. In contrast, MSFM showed the slowest degradation, providing long-term mineralization and mechanical support for osteogenesis (Figure 2G). This complementary degradation pattern enabled temporal regulation: SalB@SFH degraded rapidly in the early stages to stimulate angiogenesis, whereas MSFM degraded more gradually, supporting later-stage osteogenesis. Thus, combining the degradation characteristics of the two components allowed for coordinated progression of angiogenesis and osteogenesis, offering a promising approach for bone defect repair.

The swelling behaviors of SFH, SalB@SFH, SFM, and MSFM were also examined. MSFM demonstrated the lowest swelling rate, followed by SFM (Figure S5A), while SalB@SFH and SFH showed comparable swelling rates (Figure S5B). The reduced swelling of MSFM was attributed to the calcium-phosphate mineralization layer, which enhanced structural stability and decreased water uptake. Non-mineralized SFM absorbed more water, reflecting their higher swelling capacity. Incorporating SalB did not notably affect the swelling characteristics of SFH. Importantly, the lower swelling rate of MSFM contributes to maintaining scaffold volume stability during cranial repair, reducing the risk of excessive swelling and associated intracranial pressure.

Rheological testing further characterized scaffold performance (Figure 2H). SFH exhibited stable viscoelastic behavior, as evidenced by a higher storage modulus (G') compared to the loss modulus (G”), indicating good structural stability under shear stress. SalB@SFH showed a slightly reduced Gʹ value, particularly at low frequencies, indicating decreased crosslinking density and reduced mechanical strength. Although this mechanical weakening lowers hydrogel stability, it has biological significance: a lower elastic modulus resembling vascular matrix characteristics enhances integrin-focal adhesion signaling in HUVECs, thereby promoting cell migration and tube formation. Additionally, the looser crosslinking network accelerates SalB release, facilitating early angiogenesis.

During composite scaffold assembly, mineralized microspheres were first loaded into molds, followed by infiltration with SalB@SFH precursor solution and UV crosslinking to form a cohesive scaffold. Rheological testing revealed that SalB@SFH+MSFM exhibited significantly higher Gʹ and Gʺ compared to SalB@SFH+SFM (Figure 2H), indicating superior mechanical stability. This property is critical for maintaining scaffold integrity after implantation, thereby supporting tissue regeneration.

### Biocompatibility of composite scaffolds

The biocompatibility of the composite hydrogel scaffold was evaluated *in vitro* with BMSCs and HUVECs. BMSCs contribute significantly to osteogenesis and bone regeneration by differentiating into osteoblasts 31, while HUVECs serve as a well-established model for angiogenesis, representing the primary endothelial cell type responsible for new blood vessel formation 32. The combined use of these cell types provided a comprehensive model to assess the effects of the scaffold on both bone and vascular regeneration.

**Figure 3:**
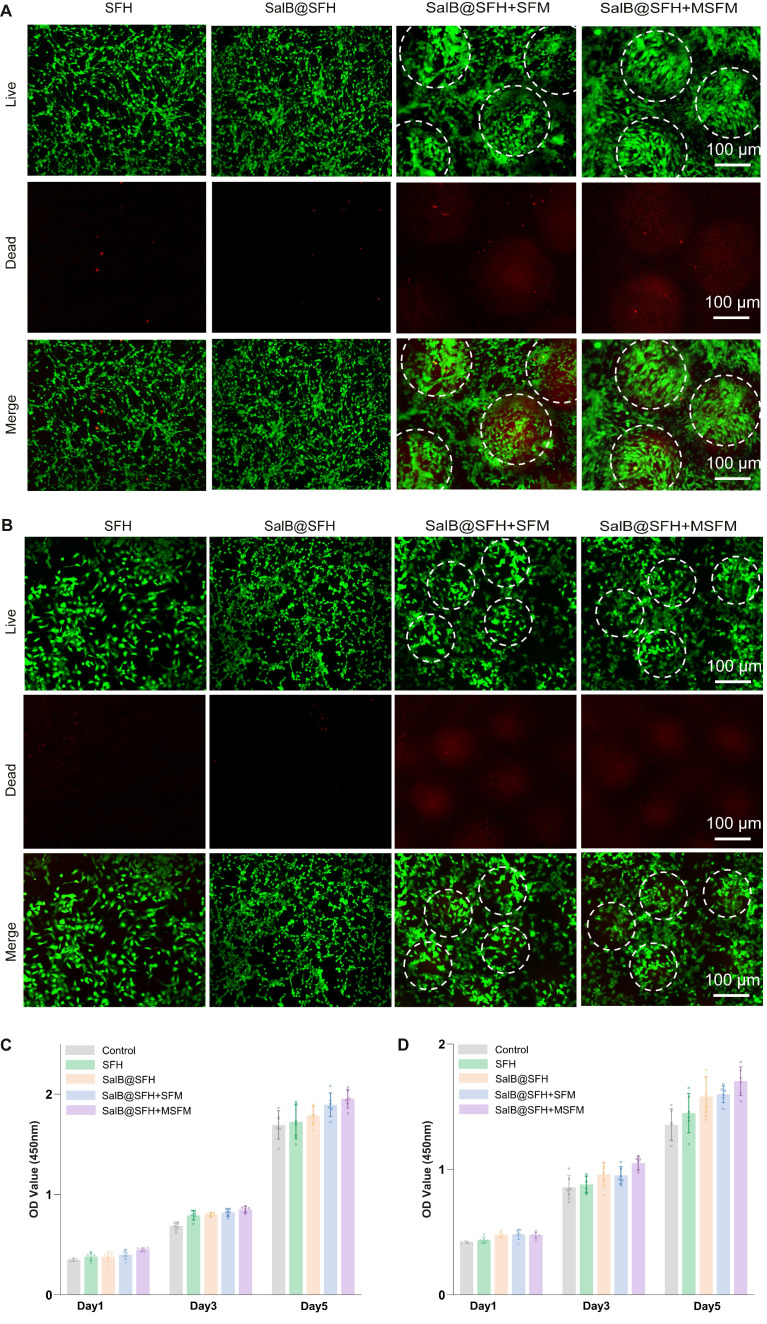
Biocompatibility of the composite hydrogel scaffold. (A) Live/dead staining of BMSCs. Scale bar = 100 μm. (B) Live/dead staining of HUVECs. Scale bar = 100 μm. (C-D) Cell viability of BMSCs and HUVECs co-cultured with composite scaffolds (*p<0.01*).

CCK-8 assays and live/dead staining were performed to evaluate scaffold cytocompatibility with BMSCs and HUVECs. CCK-8 results indicated that the composite hydrogel scaffold did not significantly inhibit cell proliferation in any group at any time point (Figures 3C-D). With increasing culture time, cell proliferation was significantly enhanced across all groups. Notably, the SalB@SFH+MSFM group exhibited a marked increase in OD values compared to the other groups (*p < 0.01*), suggesting that the combined effects of SalB and MSFM promoted BMSC proliferation. This enhancement was likely mediated by the synergistic actions of SalB, the osteoconductive properties of mineralized microspheres, and the enhanced mechanical stability of the composite scaffold. Previous studies have shown that SalB promotes BMSC proliferation and osteogenic differentiation, while also providing anti-apoptotic, anti-inflammatory, and antioxidant benefits 33. For HUVECs, cell viability was significantly higher in SalB-containing groups (SalB@SFH, SalB@SFH+SFM, and SalB@SFH+MSFM) compared with the Control and SFH groups (*p < 0.01*). The strongest effect was observed in the SalB@SFH+MSFM group. This enhancement may be attributed to calcium ion release from MSFM, which can activate calcium ion channels and downstream signaling pathways. This activation promotes endothelial cell proliferation and angiogenesis by stabilizing the cytoskeleton, improving cell adhesion, and facilitating migration 34, 35.

Cell viability and live/dead staining further validated these findings. In BMSC co-cultures, the majority of cells were viable with very few dead cells, and normal morphology was maintained (Figure 3A). Cell viability exceeded 90% across all groups, with no significant differences between them (*p > 0.05*), indicating that all scaffolds exhibited good cytocompatibility with BMSCs. In the SalB@SFH+MSFM group, BMSCs aggregated around the microspheres, suggesting that the composite scaffold promoted BMSC proliferation and metabolic activity, likely through calcium ion release and the provision of a biomimetic bone matrix that supported cell adhesion and differentiation. In HUVEC co-cultures, green fluorescence dominated with minimal red fluorescence, confirming high viability (>90%) with low cytotoxicity (Figure 3B). Compared to the Control group, HUVECs in SalB-containing groups exhibited tube-like arrangements, indicating that SalB@SFH provided a favorable microenvironment for endothelial growth, survival, and tube formation. This effect may be attributable to the scaffold architecture, cell-matrix interactions, SalB release, and possibly calcium ion release, which could enhance cytoskeletal stability and facilitate adhesion and migration.

Overall, the composite hydrogel scaffold significantly enhanced the proliferation, distribution, and functional activity of both BMSCs and HUVECs. It demonstrated excellent cytocompatibility and provided an optimal microenvironment for cell growth, highlighting its potential as a promising platform for cranial bone repair and angiogenesis in tissue engineering applications.

### *In vitro* angiogenesis ability of composite scaffolds

**Figure 4:**
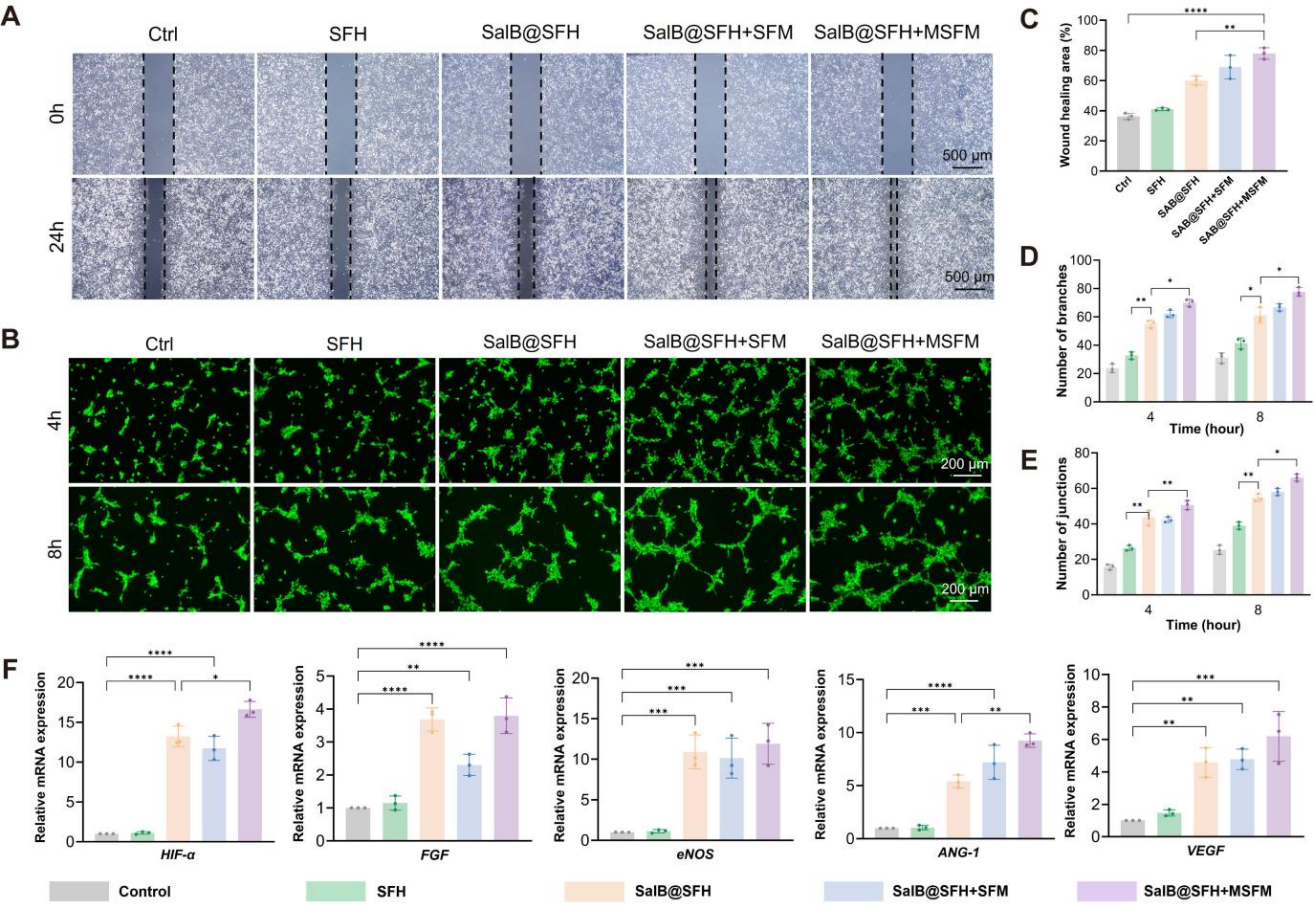
Functional evaluation of the composite scaffold in promoting angiogenesis *in vitro*. (A) Migration assay at 0 h and 24 h. Scale bar = 500 μm. (B) Schematic illustration of the angiogenesis experiment. (C-E) Quantitative analysis of migration and tube formation assays. (F) Tube formation assay at 4 h and 8 h. Scale bar = 200 μm. (G) RT-qPCR analysis of angiogenesis-related gene expression (*p < 0.001*).

The angiogenic capacity of the composite hydrogel scaffold was assessed by a Transwell co-culture system that allowed indirect contact between HUVECs and the scaffolds (Figure 4B).

In the migration assay (Figures 4A, C), the Control and SFH groups showed limited wound closure, with healing areas of 36.31% ± 2.26% and 40.96% ± 1.83%, respectively, indicating minimal stimulation of HUVEC migration. In contrast, the SalB@SFH, SalB@SFH+SFM, and SalB@SFH+MSFM groups exhibited significantly greater wound healing, with healing areas reaching 60.11%±2.06%, 68.96%±4.51%, and 77.99%±2.16%, respectively. Among them, the SalB@SFH+MSFM group achieved the largest wound closure, approaching 78%, which was markedly higher than that of the other groups (*p < 0.001*). These findings indicate that the combination of SalB and mineralized microspheres exerted a synergistic effect in promoting endothelial cell migration. This enhancement is likely associated with the pro-angiogenic property of SalB together with calcium ion release from mineralized microspheres, which regulates cytoskeletal remodeling, strengthens adhesion, and facilitates endothelial motility 36, 37.

Further validation of the pro-angiogenic potential of the composite hydrogel scaffold was obtained through the tube formation assay. In the Control and SFH groups, vascular networks were sparse, with few branches and connection points. By contrast, the SalB@SFH and SalB@SFH+SFM groups formed more robust vascular networks, while the SalB@SFH+MSFM group displayed the densest and most complex networks, indicating the most pronounced angiogenic effect (Figure 4F). Quantitative analysis showed a substantial increase in branch and junction formation in the SalB@SFH, SalB@SFH+SFM, and SalB@SFH+MSFM groups in comparison to the Control and SFH groups (*p < 0.01*) (Figures 4D, E). The inclusion of mineralized microspheres further enhanced angiogenesis, likely through calcium ion release. Calcium signaling regulates endothelial cell proliferation and contributes to vascular lumen stabilization during angiogenesis 37.

Further evidence was obtained from RT-qPCR analysis of angiogenesis-related genes, including *HIF-α, VEGF, ANG-1, ENOS,* and *FGF* 38-42. The transcriptional levels of these genes were significantly lower in the Control and SFH groups, although slightly higher in SFH, possibly due to the lower stiffness of hydrogels compared to standard tissue culture plates. In contrast, SalB@SFH, SalB@SFH+SFM, and SalB@SFH+MSFM scaffolds induced significant upregulation of these genes (Figure 4G). The SalB@SFH+MSFM group demonstrated the highest expression levels, with *HIF-α, VEGF, ANG-1, eNOS,* and *FGF* increasing by 13.8-, 5.2-, 8.5-, 9.6-, and 3.1-fold, respectively, compared with control group (*p < 0.01*). The results demonstrate that the SalB@SFH+MSFM scaffold promotes angiogenesis through mechanisms including hypoxia-mimetic signaling and NO-mediated endothelial function enhancement. This aligns with the temporal regulatory design of the scaffold, in which SalB primarily drives early-stage vascularization while MSFM supports later-stage osteogenesis, and underscores the ability of the scaffold to optimize the vascular microenvironment through multi-target regulation.

In summary, the composite hydrogel scaffold demonstrated robust pro-angiogenic activity by facilitating endothelial cell migration, promoting tube formation, and upregulating angiogenesis-related genes. Its capacity to sequentially and synergistically promote angiogenesis and osteogenesis emphasizes its potential as a multifunctional approach for regenerative medicine and tissue repair.

### *In vitro* osteogenesis ability of composite scaffolds

**Figure 5:**
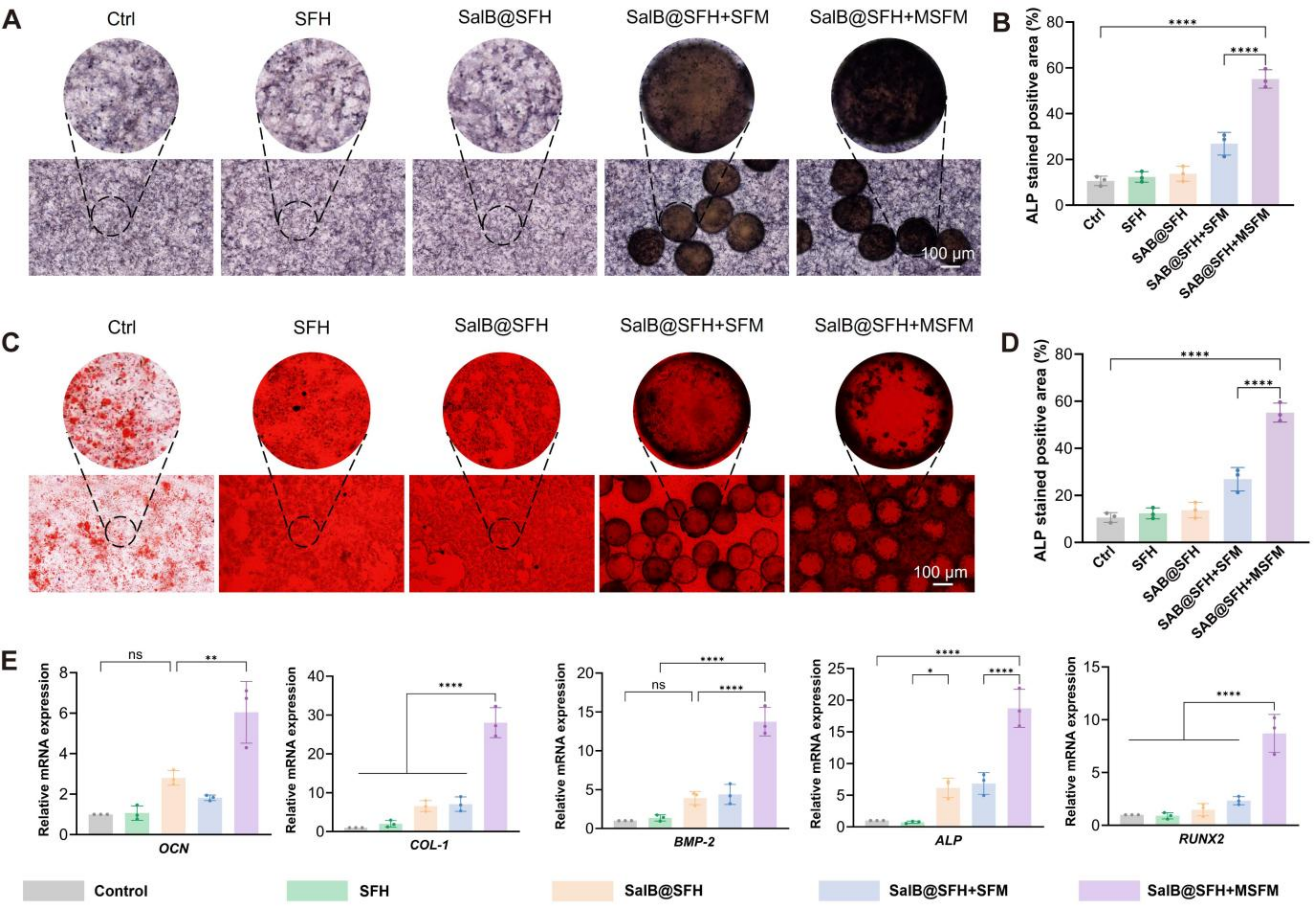
Functional evaluation of the composite scaffold in promoting osteogenesis *in vitro*. (A-B) ALP staining and subsequent quantitative analysis at day 7. Scale bar = 100 μm. (C-D) ARS staining and subsequent quantitative analysis at day 7. Scale bar = 100 μm. (E) Quantitative PCR analysis of osteogenesis-related gene expression (*p < 0.001*).

The composite hydrogel scaffold combining SalB@SFH and MSFM showed significant potential for promoting osteogenesis. ALP staining at day 7 was selected as a key indicator of early osteogenic differentiation, since ALP is critical for bone matrix synthesis and mineralization at this stage 43. On day 7, the SalB@SFH+MSFM group exhibited markedly stronger ALP intensity than all other groups, with prominent purple coloration indicative of active osteogenic differentiation (Figure 5A). Quantitative analysis confirmed that the ALP-positive area in the SalB@SFH+MSFM group was notably larger than in the Control group (*p < 0.001*) (Figure 5B). These results suggest that mineralized microspheres made an important contribution to early osteogenesis. Although the SalB@SFH+SFM group also showed enhanced ALP staining, its intensity was lower than that of the SalB@SFH+MSFM group. The improved performance of the MSFM-containing scaffold can be ascribed to the dual functionality of mineralized microspheres, which not only enhanced mechanical stability but also directly participated in osteogenesis through calcium phosphate components on their surface, thereby promoting BMSC differentiation. In contrast, non-mineralized microspheres mainly strengthened the mechanical properties of the scaffold, creating a more conducive environment for the proliferation and differentiation of cells, but lacking the direct osteogenic stimulation provided by MSFM.

To assess late-stage osteogenesis and mineralization, ARS staining was performed on day 14. This method is commonly used to assess calcium deposition, and the 14-day time point reflects the later stages of osteogenesis, when matrix mineralization becomes prominent 44. The SalB@SFH+MSFM group exhibited the strongest staining intensity, with vivid red coloration indicating extensive calcium deposition and mineralization (Figures 5C-D). Quantitative analysis showed significantly greater calcium deposition in this group compared with all others. Although the remaining groups also demonstrated increased staining relative to the Control, their intensity was weaker than that of the SalB@SFH+MSFM. These findings confirm that mineralized microspheres enhanced not only early osteogenic differentiation but also late-stage mineralization. In contrast, non-mineralized microspheres primarily provided mechanical reinforcement, providing scaffold stability to facilitate cell adhesion and proliferation, but lacked the mineralization-promoting effect necessary for efficient calcium deposition.

RT-qPCR analysis of genes related to osteogenesis, including *ALP, COL-1, BMP-2, RUNX2, and OCN* 45-49*,* further elucidated the molecular mechanisms underlying these effects (Figure 5E). These genes were selected as key markers to capture distinct stages of osteogenic differentiation and matrix formation. The SalB@SFH+MSFM group exhibited the highest expression levels with *ALP*, *COL-1*, *BMP-2*, *RUNX2*, and *OCN* upregulated by approximately 4.8-, 6.3-, 9.6-, 4.3-, and 5.8-fold, respectively, compared with the Control (*p < 0.001*). These results indicate that the SalB@SFH+MSFM scaffold markedly promoted osteogenic differentiation by enhancing bone matrix synthesis and mineralization. In comparison to the SFH and Control groups, the SalB@SFH+SFM group demonstrated elevated expression of osteogenesis-related genes, confirming that non-mineralized microspheres contributed to osteogenic differentiation by improving the microenvironment and mechanical properties of the scaffold. Nevertheless, the SalB@SFH+SFM group exhibited lower gene expression compared to the SalB@SFH+MSFM group, indicating that the absence of bioactive mineral components limited its capacity to directly stimulate osteogenesis. In summary, the inclusion of mineralized microspheres significantly enhanced both early and late stages of osteogenesis, facilitating effective bone defect repair through dual regulation of osteogenic differentiation and matrix mineralization.

### RNA transcriptome sequencing analysis

**Figure 6:**
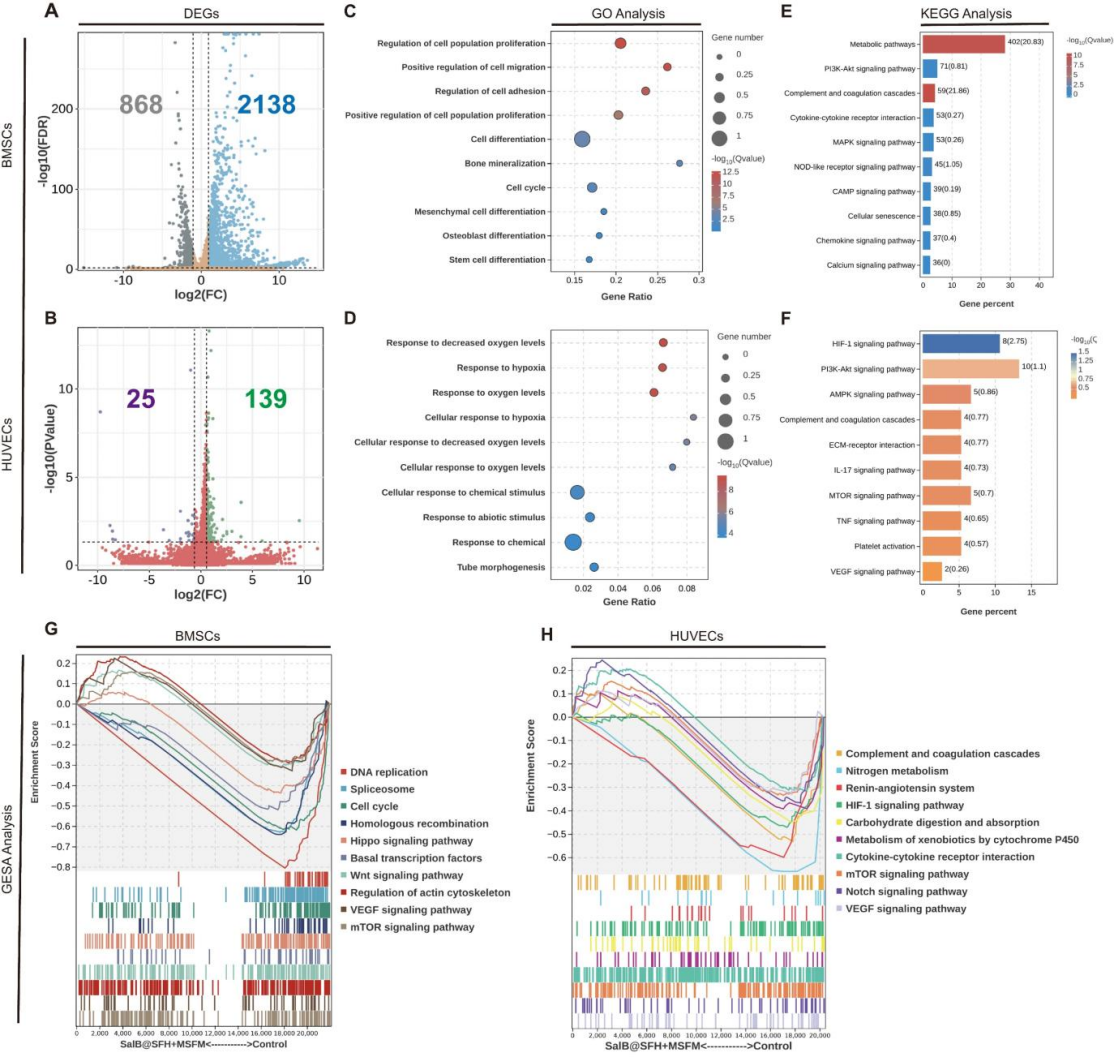
Transcriptome sequencing analysis of BMSCs and HUVECs. (A-B) Differentially expressed genes (DEGs) between the Control and SalB@SFH+MSFM groups. (C-D) GO enrichment analysis of DEGs. (E-F) KEGG pathway enrichment analysis of DEGs. (G-H) GSEA of DEGs.

To explore the molecular mechanisms driving the osteogenic and angiogenic effects of the composite scaffold, transcriptomic sequencing was performed using BMSCs and HUVECs co-cultured with the scaffold compared with controls. In BMSC, 2,138 genes were upregulated and 868 genes were downregulated, while in HUVECs, 139 genes were upregulated and 25 genes were downregulated (Figure 6A, B).

GO, KEGG, and GSEA analyses of BMSCs (Figures 6C, D) revealed synergistic activation of pathways involved in proliferation, including cell cycle and PI3K-Akt/MAPK, together with osteogenic differentiation networks such as Runx2 and BGLAP. The PI3K-Akt/MAPK pathway plays a crucial role in cell survival, proliferation, and differentiation 50. It is activated by growth factors and cytokines, including VEGF 51, and is involved in the regulation of both angiogenesis and osteogenesis. Activation of this pathway therefore contributes to the coupling of angiogenesis with osteoblast differentiation and mineralization, supporting coordinated tissue regeneration.

In HUVECs, the scaffold activated hypoxia response pathways (HIF-1α/VEGF axis), tube formation, and extracellular matrix (ECM) receptor interaction networks (Figures 6E, F). The HIF-1α/VEGF pathway regulates migration of endothelial cells and angiogenesis 52. Under hypoxic conditions, HIF-1α activation leads to VEGF upregulation, which promotes endothelial cell proliferation, migration, and tube formation. This angiogenic process also creates a favorable microenvironment for osteogenesis by improving oxygen and nutrient delivery to regenerating tissue.

Collectively, transcriptome data indicate that the scaffold promotes osteogenic differentiation of BMSCs and enhances angiogenesis of HUVECs through multi-pathway regulation. These findings highlight the dual regulatory capacity of the scaffold: driving osteogenesis through metabolic-immune-stemness while coordinating angiogenesis through hypoxia-ECM-chemokine pathways, thereby providing a biomimetic strategy for integrated osteo-vascular regeneration.

### *In vivo* assessment of osteogenesis

**Figure 7:**
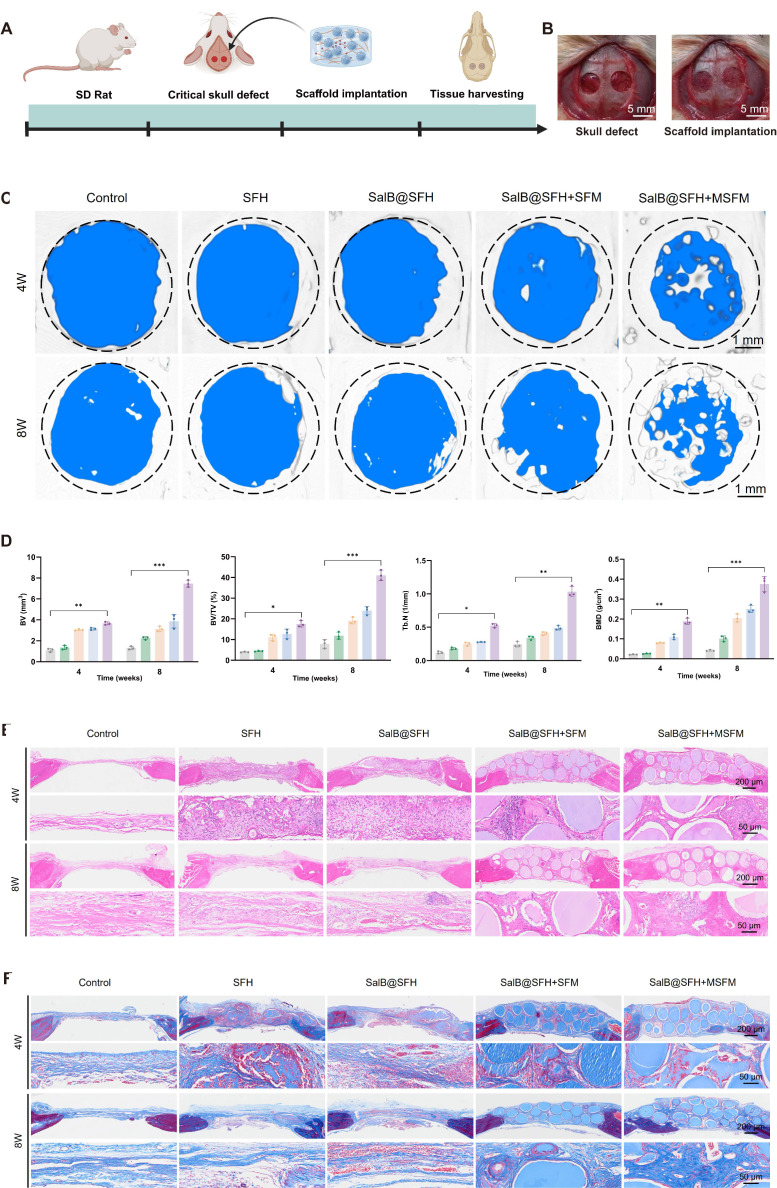
Micro-CT and histological analysis of cranial defect repair. (A) Protocol for establishing the cranial defect model. Scale bar = 5mm. (B) Surgical schematic of cranial defects in rats. (C-D) Micro-CT reconstruction and quantitative analysis of rat skull samples at 4 and 8 weeks. Scale bar = 1mm. (E-F) H&E and Masson's trichrome staining of regenerated bone tissue. Scale bar = 200μm and 50 μm.

To assess the therapeutic effects of the composite scaffold *in vivo*, a 5-mm cranial defect model was established in 5- to 6-week-old SD rats (Figures 7A, B). Micro-CT reconstruction revealed that, at 4 and 8 weeks, the SalB@SFH+SFM group showed a significantly higher new BV compared to the Control and SFH groups. The SalB@SFH+MSFM group demonstrated the most prominent bone regeneration, characterized by abundant new bone formation at both the margins and central areas (Figure 7C). Quantitative analysis confirmed that the SalB@SFH+SFM and SalB@SFH+MSFM groups significantly outperformed the Control and SFH groups in BV, BV/TV, Tb.N, and BMD (Figure 7D) (*p < 0.0001*). These results indicate that MSFM contributed to bone regeneration and the incorporation of SalB further enhanced bone formation, with both components acting synergistically to promote bone repair.

Histological analysis using H&E staining revealed structural changes during cranial defect repair. At both 4 and 8 weeks, the Control group exhibited persistent defects with minimal new bone formation restricted to the margins. In contrast, the SalB@SFH and MSFM groups displayed enhanced bone regeneration and more extensive new bone formation (Figure 7E). The H&E staining sections revealed active bone remodeling with evidence of mineralization and formation of trabecular structures. The SalB@SFH+MSFM group exhibited the greatest improvement, with compact new bone, increased trabecular proliferation, and higher bone density, signifying more effective defect repair.

Masson's Trichrome staining 53 further highlighted differences in collagen fiber organization and bone matrix remodeling among the experimental groups (Figure 7F). Sparse and disorganized collagen fibers were observed in the Control group, reflecting inadequate bone matrix support and limited bone regeneration. In contrast, the SalB@SFH+MSFM group exhibited abundant, well-aligned collagen fibers that were seamlessly integrated with the surrounding bone, confirming more effective bone matrix formation. This outcome is attributable to the synergistic effects of mineralized microspheres and SalB, which not only supported osteogenesis but also promoted the formation of a structurally organized collagen matrix essential for bone healing. These results underscore the complementary contributions of mechanical stability and bioactive signaling in facilitating bone matrix formation, which is critical for effective cranial defect repair. In particular, the ability of the scaffold to support both mineralization and collagen organization enhances the stability and functionality of regenerated bone, highlighting its promise as an effective solution for bone defect repair.

**Figure 8:**
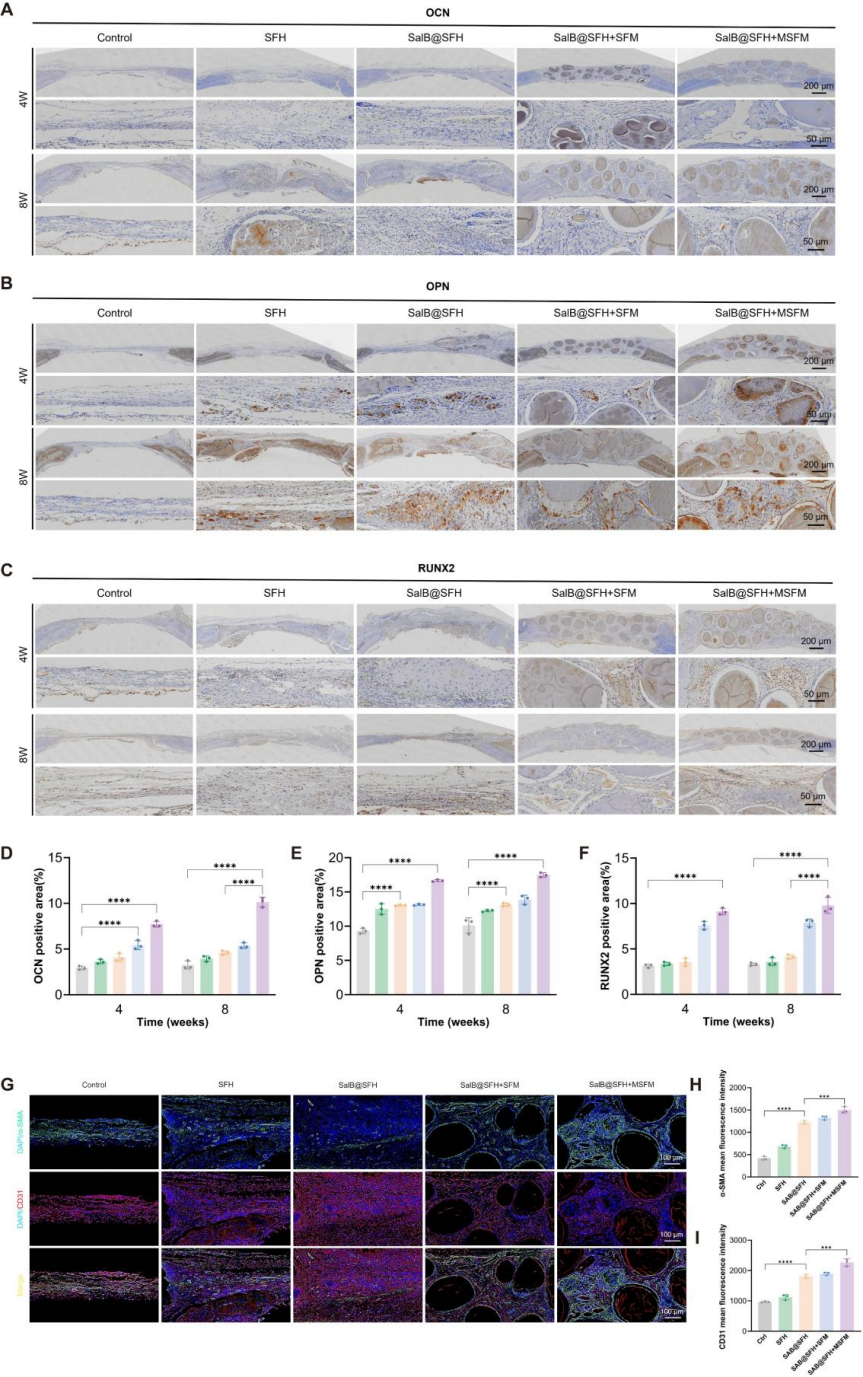
*In vivo* histological analysis of cranial defect repair. (A-C) Immunohistochemical staining of OCN, OPN, and RUNX2 at 4 and 8 weeks. Scale bar = 200 μm and 50 μm. (D-F) Quantitative analysis of OCN, OPN, and RUNX2 expression by immunohistochemistry. (G-H) Immunofluorescence staining and quantitative analysis of CD31 and α-SMA at 4 weeks. Scale bar = 100 μm.

The expression of key osteogenic markers was evaluated using immunohistochemistry and immunofluorescence staining, shedding light on the bone regeneration process. OCN, OPN, and RUNX2 were selected as essential osteogenic markers. OCN is critical for regulating bone mineralization 54, OPN contributes to osteoblast proliferation and differentiation 55, and RUNX2 functions as a master transcription factor for osteogenic differentiation 56. At both 4 and 8 weeks, the Control group showed sparse tissue with poorly organized connective structures. In some areas, the surrounding tissue was detached from the defect margin, reflecting the limited capacity for natural healing without intervention. The SalB@SFH+MSFM group exhibited strong positive staining for OCN, OPN, and RUNX2, indicating active bone formation and maturation (Figures 8A-C). Quantitative analysis showed markedly higher expression of these markers in the SalB@SFH+MSFM group compared with the Control group (Figures 8D-F). The positively stained regions were densely distributed, and the newly formed bone tissues showed close continuity with the defect edges, demonstrating effective osteogenesis and tissue integration. The robust expression of these osteogenic markers in the SalB@SFH+MSFM group suggests that the combination of SalB and mineralized microspheres supported osteoblast differentiation and enhanced bone matrix deposition, contributing to the expedited bone repair. These outcomes are critical for effective bone defect repair. Overall, the SalB@SFH+MSFM composite scaffold facilitated osteoblast recruitment, enhanced osteogenic differentiation, and promoted ECM formation, providing mechanistic evidence for its role in bone tissue engineering.

Immunofluorescence staining at week 4 further confirmed enhanced angiogenesis in the SalB@SFH+MSFM group. Strong red fluorescence for CD31 (endothelial cells) and green fluorescence for α-SMA (smooth muscle cells) indicated active neovascularization (Figures 8G, H). The SalB@SFH+MSFM group exhibited significantly more blood vessels than the other groups, suggesting that the scaffold can establish a pro-angiogenic microenvironment. Enhanced vascularization is crucial for delivering oxygen and nutrients to regenerating bone tissue, thereby supporting overall repair. This improvement likely resulted from the combined activity of SalB, which promoted endothelial cell proliferation, and mineralized microspheres, which provided structural support for vascular stabilization. Together, these effects enhanced both angiogenesis and osteogenesis, underscoring the potential of the SalB@SFH+MSFM scaffold as a multifunctional material for bone repair.

### *In vivo* biocompatibility

**Figure 9:**
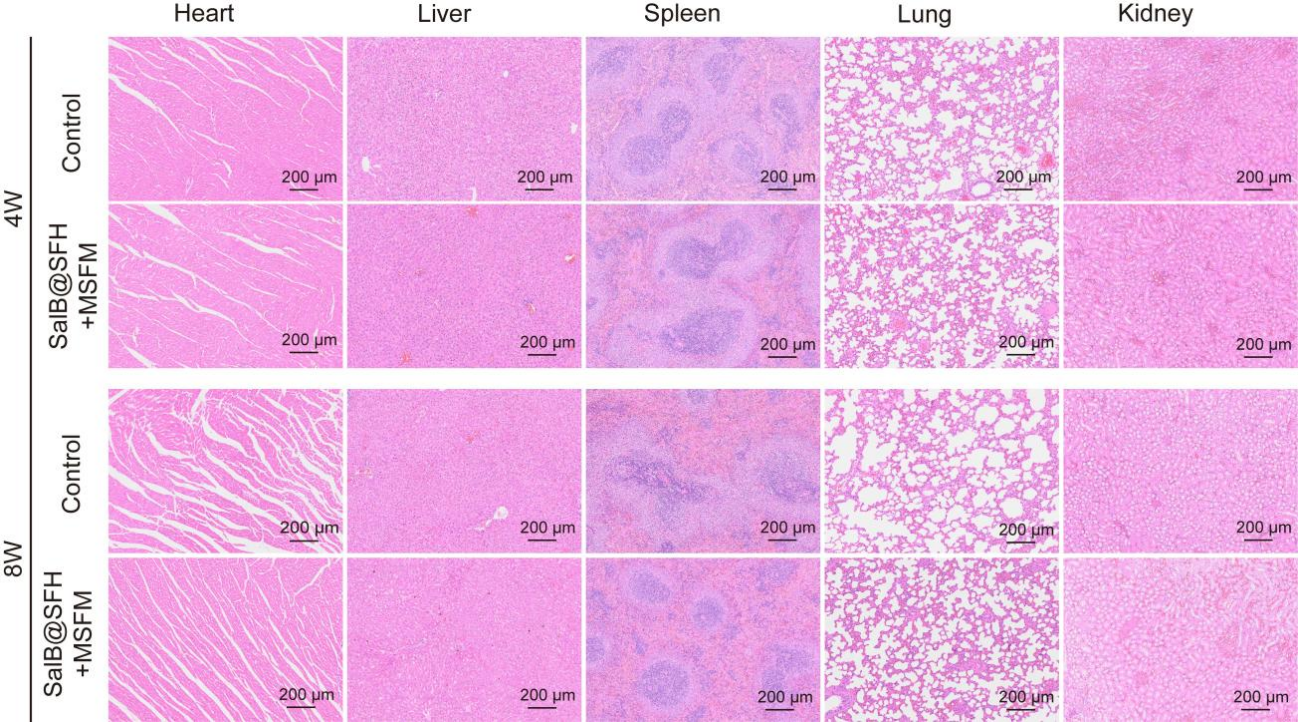
*In vivo* biocompatibility assessment. H&E staining of organ sections (heart, liver, spleen, lungs, and kidneys) from rats in each experimental group. Scale bar = 200 μm.

Histological analysis of key organs (heart, liver, spleen, lungs, and kidneys) stained with H&E (Figure 9) showed no pathological abnormalities or adverse effects. These results indicate that the scaffold was compatible *in vivo* and did not induce systemic toxicity, highlighting its potential for future translational applications.

## Conclusion

A temporally functional composite hydrogel scaffold was developed for cranial defect repair by integrating SalB@SFH with MSFM. This system achieved sequential modulation of angiogenesis and osteogenesis through differential degradation kinetics and controlled release of bioactive components. SalB@SFH enabled the sustained release of SalB, which significantly promoted endothelial cell migration, capillary-like tube formation, and angiogenesis-related gene expression. Early-stage angiogenesis was primarily driven through the HIF-1α/VEGF signaling pathway, supporting cell proliferation and vascular network formation.

The resulting vascularization provided a favorable microenvironment for osteogenesis by delivering oxygen, nutrients, and growth factors essential for osteoblast differentiation and bone matrix deposition. The mineralized microspheres further enhanced regeneration by releasing calcium ions to stabilize endothelial cells and support vessel maturation, while simultaneously providing mechanical reinforcement and mineralization nuclei to facilitate calcium-phosphate deposition. This dual contribution promoted osteoblast differentiation, matrix mineralization, and structural stability. *In vivo* findings showed that the composite scaffold enhanced vascularized bone regeneration in cranial defects, with Micro-CT and histological analyses confirming significant improvements in bone volume, trabecular architecture, and collagen organization.

This work presents a biomimetic strategy that coordinates vascularization and mineralization, overcoming the limitations of static repair materials. By mimicking the natural bone healing cascade, the scaffold offers a clinically translatable solution for complex cranial defects. The temporal regulation of bioactive molecule release and mineralization within a single scaffold represents an innovative approach to functional bone repair. Sequential regulation of angiogenesis and osteogenesis positions this scaffold holds great promise for clinical use in cranial bone regeneration.

Despite these promising results, limitations remain. The rat cranial defect model may not fully recapitulate the human cranial environment; larger animal such as rabbits or pigs are necessary to better assess clinical applicability and safety. Further optimization of degradation kinetics and mechanical properties will also be crucial for successful clinical translation.

Overall, this scaffold holds significant potential for clinical use in cranial defect repair. Its ability to modulate both angiogenesis and osteogenesis in a spatiotemporal manner provides a versatile platform for regenerative medicine 57, 58. The principles established in this study may extend to other areas of tissue engineering, including the regeneration of bone, cartilage, and vascular tissues, where precise control over sequential healing processes is crucial 59.

## Supplementary Material

Supplementary figures and tables.

## Figures and Tables

**Figure 1:**
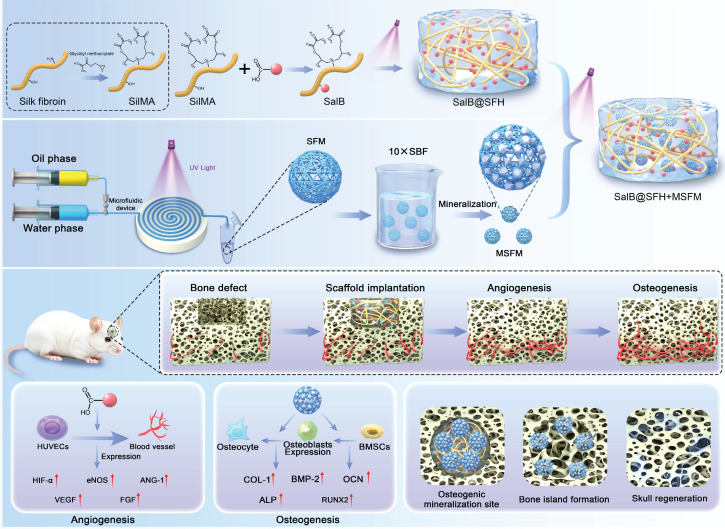
Schematic illustration of the temporally regulated hydrogel composite scaffold designed for cranial bone defect repair through sequential promotion of angiogenesis and osteogenesis.
