# Supplementary material for: A temporally functional composite hydrogel scaffold for cranial defect repair via sequential modulation of angiogenesis and osteogenesis
Source: Theranostics. 2026 Jan 1;16(3):1445–65. doi: 10.7150/thno.119835 (PMC12679569; doi:10.7150/thno.119835)
Supplement: Supplementary file 1 — Supplementary figures and tables. [file thnov16p1445s1.pdf]

Supplementary Materials for

A Temporally Functional Composite Hydrogel Scaffold for Cranial Defect Repair via Sequential Modulation of Angiogenesis and Osteogenesis

Zongqiang Lv, Bo Sun, Rong Li, Bowen Zhao, Hongxiang Wang, Ning Luo, Xin Ding , Xuan Tang, Chunlin Wang*, Long Bai*, Jiacan Su*, Juxiang Chen*

*Corresponding authors at:
Department of Neurosurgery, No.901 Hospital of Joint Logistics Support Force of PLA, Hefei, 230031, Anhui, China
E-mail addresses: sprlin1105@126.com (Chunlin Wang)

*Corresponding authors at:
Organoid Research Center, Institute of Translational Medicine, Shanghai University, Shanghai, 200444, China
E-mail addresses: drsujiacan@163.com (Jiacan Su), bailong@shu.edu.cn (Long Bai)

*Corresponding author at:
Department of Neurosurgery, The First Affiliated Hospital of Naval Medical University, Shanghai 200433, China
E-mail addresses: juxiangchen@smmu.edu.cn (Juxiang Chen)

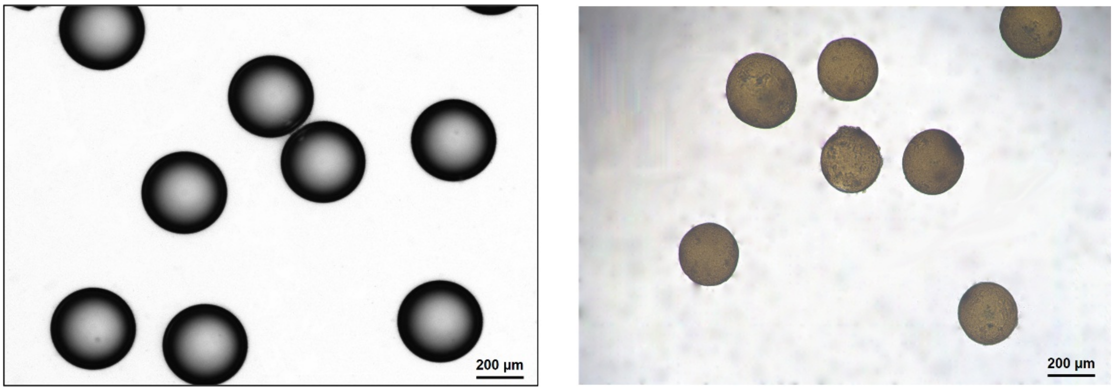

A **B**

Figure S1. Morphology of hydrogel microspheres. (A) Bright-field image of hydrogel microspheres. (B) Optical microscopy image of mineralized hydrogel microspheres. Scale bar = 200 μm.

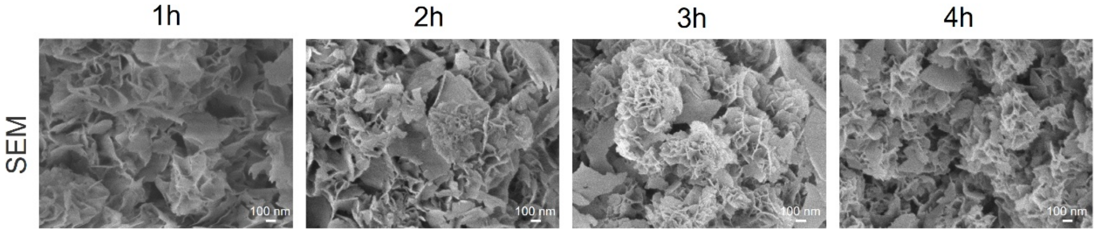

Figure S2. SEM images of MSFM at 1 h, 2 h, 3 h, and 4 h. Scale bar = 100 nm.

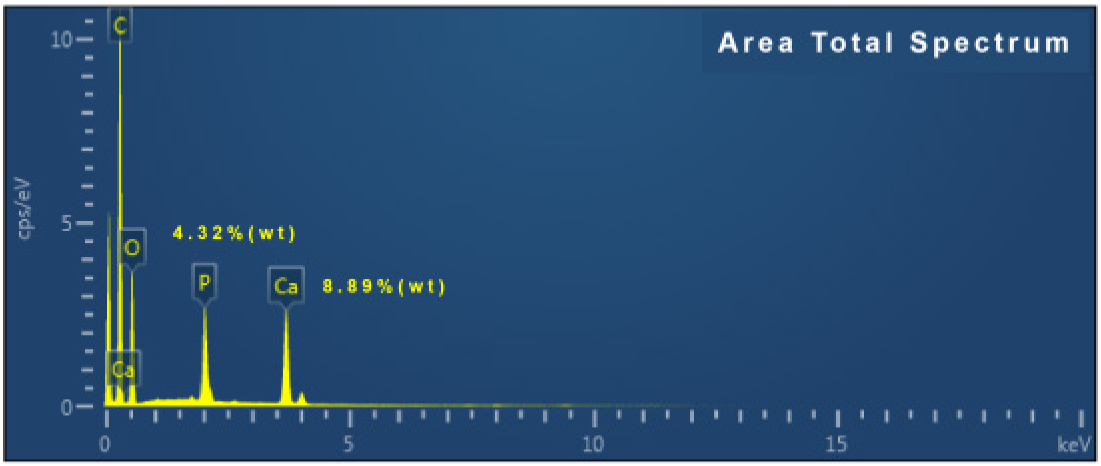

Figure S3. EDS area spectrum of mineralized hydrogel microspheres.

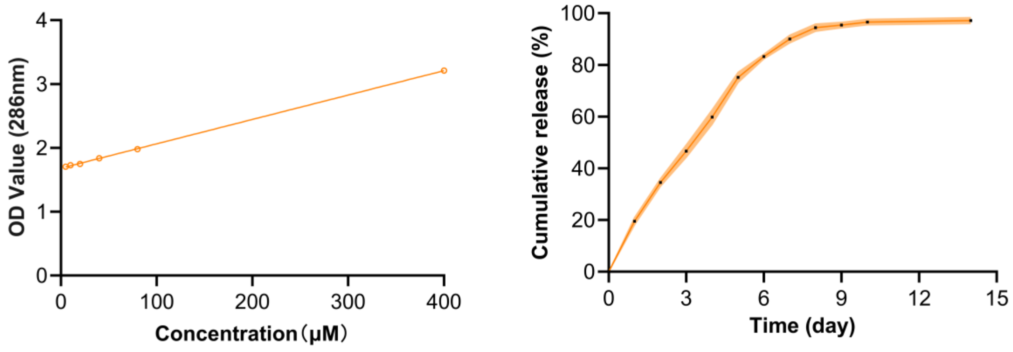

A **B**

Figure S4. Characterization of SalB release from hydrogel. (A) Standard calibration curve of SalB. (B) Release profile of SalB from hydrogel.

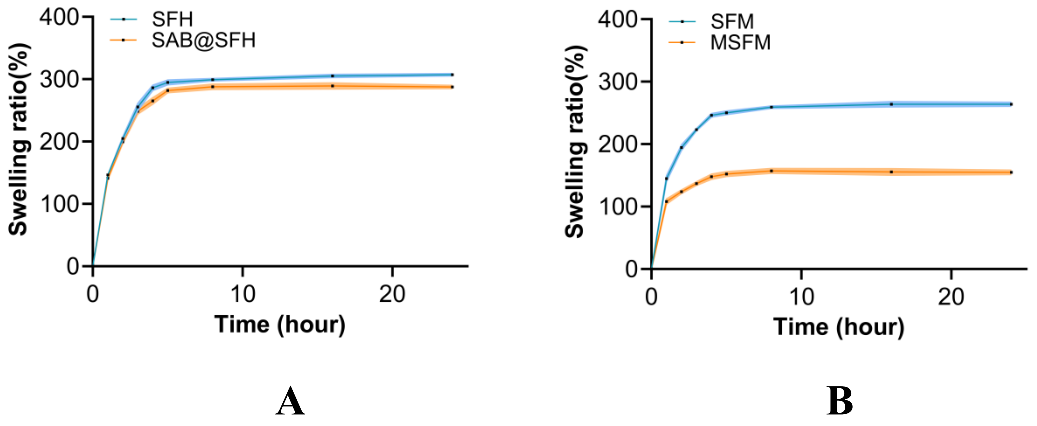

Figure S5. Swelling behavior of hydrogels and hydrogel microspheres. (A) Swelling curves of SFH and SalB@SFH. (B) Swelling curves of SFM and MSFM.

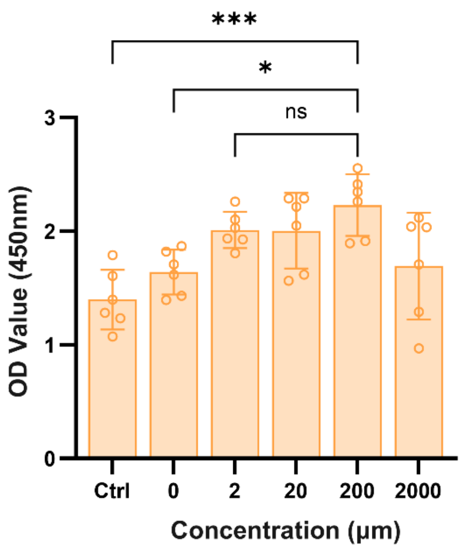

Figure S6. Effective concentration of SalB in SalB@SFH.

Table S1. Preparation of 10× SBF.

**Reagent**	**Order**	**Amount (g)**	**Concentration(mM)**
NaCl	1	116.8860	1000
KC1	2	0.7456	5
$CaCl_2$:$2H_2O$	3	7.3508	25
$MgCl_2$:$6H_2O$	4	2.0330	5
NaH_2PO_4	5	2.3996	10

Table S2. Primer sequences used for RT-qPCR.

Gene symbol	5’-3’
HIF1A-S	GCTCATCAGTTGCCACTTCCAC
HIF1A-A	CCAAATCACCAGCATCCAGAAG
VEGFA-S	AGGGCAGAATCATCACGAAGT
VEGFA-A	GCACACAGGATGGCTTGAAGA
FGF2-S	GAGAAGAGCGACCCTCACATCA
FGF2-A	GTTCGTTTCAGTGCCACATACC
VWF-S	TGGAAGCATCACCACCATTGAC
VWF-A	AAGTCAAGTATCGCACAGCAAAG
NOS3-S	AGACAAGGCAGCAGTGGAAATC
NOS3-A	TCTGCTCATTCTCCAGGTGCTT
ANG1-S	CAGGAGGATGGTGGTTTGATG
ANG1-A	TAGTGCCACTTTATCCCATTCAG
ALP-S	GGCACCTGCCTTACCAACTCT
ALP-A	GTTGTGGTGTAGCTGGCCCTTA
OCN-S	GGAGGGCAATAAGGTAGTGAACAG
OCN-A	ATAGCTCGTCACAAGCAGGGT
RUNX2-S	ATGACACTGCCACCTCTGACTTCT
RUNX2-A	AGGGATGAAATGCTTGGGAACT
BMP2-S	GCCCATTTAGAGGAGAACCCAG
BMP2-A	TTCTCGTTTGTGGAGCGGAT
COL-S	CTCCCAGAACATCACCTATCACT
COL-A	GGAGGTCTTGGTGGTTTTGTATT
VEGFA-S	GTAACGATGAAGCCCTGGAGTG
VEGFA-A	TCACAGTGAACGCTCCAGGAT
GAPDH-S	CCTCGTCCCGTAGACAAAATG
GAPDH-A	TGAGGTCAATGAAGGGGTCGT
